# Distributed Target Detection in Unknown Interference

**DOI:** 10.3390/s22072430

**Published:** 2022-03-22

**Authors:** Kaiming Xu, Yunkai Deng, Zhongjun Yu

**Affiliations:** 1 The Aerospace Information Research Institute, Chinese Academy of Sciences, Beijing 100094, China; ykdeng@mail.ie.ac.cn (Y.D.); yuzj@aircas.ac.cn (Z.Y.); 2 The School of Electronic, Electrical and Communication Engineering, University of Chinese Academy of Sciences, Beijing 101408, China

**Keywords:** multichannel signal, distributed target, unknown interference, generalized likelihood ratio test, Rao test, Wald test

## Abstract

Interference can degrade the detection performance of a radar system. To overcome the difficulty of target detection in unknown interference, in this paper we model the interference belonging to a subspace orthogonal to the signal subspace. We design three effective detectors for distributed target detection in unknown interference by adopting the criteria of the generalized likelihood ratio test (GLRT), the Rao test, and the Wald test. At the stage of performance evaluation, we illustrate the detection performance of the proposed detectors in the presence of completely unknown interference (not constrained to lie in the above subspace). Numerical examples indicate that the proposed GLRT and Wald test can provide better detection performance than the existing detectors.

## 1. Introduction

Multichannel signal detection is a fundamental problem in the signal processing community [1,2,3,4,5,6,7,8]. Kelly first derived the generalized likelihood ratio test (GLRT) for multichannel radar systems in unknown noise in 1986 [9]. Then, many other detectors were proposed, such as the adaptive matched filter (AMF) [10], the adaptive coherence estimator (ACE) [11], and so on. Besides the possible target, there is often interference [12]. In [13], the problem of distributed target detection in the presence of known interference was considered, and several detectors were proposed according to the GLRT criterion. The interference in [13] is known in the sense that the interference lies in a known subspace but its coordinate is unknown. The related Rao tests were derived in [14], while the point-target case of the Wald test was given in [15]. Recently, it was pointed out in [16] that the detectors proposed in [14,15] are essentially derived according the variations of the Rao test and Wald test, respectively. According to the criteria of the GLRT, the Rao test, and the Wald test, three detectors were proposed in [17] for the problem of point target detection in a kind of partially known interference, called orthogonal interference therein. It was shown in [18] that the GLRT in [17] can also be derived according to the criterion of gradient test.

In fact, the operating environment of the radar system usually changes rapidly. Hence, there may be no reliable prior information about the interference. In [19], two detectors were proposed according to the GLRT and the Wald test for the problem of point target detection in the presence of completely unknown interference, which can be caused by static objects or a hostile jammer. The statistical performance of the GLRT in [19] was investigated in [20]. It was shown in [16] that both the Rao and Wald tests coincided with the GLRT. Moreover, It was pointed out in [16] that the Wald test proposed in [19] was essentially a variation of the Wald test.

Note that with the improvement of radar resolution, a target usually occupies successive multiple range bins. In other words, a target is usually a distributed target [21,22]. However, to the best of our knowledge, no effective detector has been proposed for the problem of detecting a distributed target in completely unknown interference. To bridge this gap, in this study, we consider the problem of distributed target detection in the presence of completely unknown interference. We adopt the criteria of the GLRT, the Rao test, and the Wald test to design detectors, because there is no optimum detector. This is usually called the uniformly most powerful (UMP) test (There is no UMP test, due to the fact that the interference and noise covariance matrix are both unknown.). We evaluate the detection performance of both of the proposed detectors through simulation data. The results show that the proposed detectors according to the GLRT and the Wald test can provide higher probabilities of detection (PDs) than the existing detectors.

## 2. Problem Formulation

Assume that a radar system has *N* antennas. A distributed target, if present, occupies *K* successive range bins. Then, the test data reflected by the distributed target can be denoted by an N×K matrix X. Under the null hypothesis $H_{0}$, X contains noise W and completely unknown interference Q. In contrast, under the alternative hypothesis $H_{1}$, X contains noise W, completely unknown interference Q, and signal H, which is assumed to lie in a known subspace spanned by an N×p full-column-rank matrix A. Hence, we have H=AB, where the p×K matrix denotes the coordinate of the signal. We adopt the method in [19] to model the completely unknown interference at the stage of detector design. Precisely, it is temporally assumed that Q lies in a subspace orthogonal to the signal subspace. Hence, we have Q=JD, where J is an N×(N−p) full-column-rank matrix satisfying (We use (1) to constrain the unknown interference mainly for mathematical tractability. The constraint in (1) is abandoned at the stage of detection performance evaluation.)

(1) $$A^{H}J=0_{p\times (N-p)},$$

with ${\left(\cdot \right)}^{H}$ denoting the conjugate transpose; D is an unknown (N−p)×K matrix. In practice, the noise covariance matrix of W, denoted as R, is unknown. A common method to overcome this problem is using training data, usually collected in the vicinity of the test data. Assume that there are *M* training data, $x_{m}$, m=1,2,…,M, sharing the same noise covariance matrix R. To summarize, we have the following binary hypothesis test:

(2) $$\left\{\begin{array}{c} H_{0}:X=JD+W,\;\;X_{M}=W_{M},\\ H_{1}:X=AB+JD+W,\;\;X_{M}=W_{M}, \end{array}\right.$$

where $X_{M}=\left[x_{1},x_{2},\ldots ,x_{M}\right]$, and $W_{M}$ is the noise matrix in $X_{M}$ (It seems that the detection problem in (2) is similar to that in [13,14]. However, there is an essential difference. The interference matrix J in (2) can be obtained by the singular valued decomposition of A. In other words, no prior information about the interference is needed for (2). In contrast, the interference subspace needs to be set in advance in [13,14]).

## 3. Detector Derivations

Since there is no optimum detector for the detection problem in (2), we adopt the criteria of the GLRT, the Rao test, and the Wald test to design detectors and then compare their detection performance in the next section.

### 3.1. GLRT

The GLRT can be expressed as

(3) $$t_{\mathrm{GLRT}}=\frac{\max_{B,D,R}\;f_{1}\left(X,X_{M}\right)}{\max_{D,R}\;f_{0}\left(X,X_{M}\right)},$$

where $f_{1}\left(X,X_{M}\right)$ and $f_{0}\left(X,X_{M}\right)$ are the joint probabilty density functions (PDFs) of X and $X_{M}$ under Hypotheses $H_{1}$ and $H_{0}$, respectively. To obtain the GLRT for (2), we can adopt the mathematical derivations similar to that in [13]. For simplicity, we omit the derivation procedure and just list the resulting GLRT

(4) $$t_{\mathrm{GLRT}}=\det\left(I_{K}+{\tilde{X}}^{H}P_{\tilde{J}}^{⊥}\tilde{X}\right),$$

where $\tilde{X}=S^{-\frac{1}{2}}X$, $\tilde{J}=S^{-\frac{1}{2}}J$, $S=X_{M}X_{M}^{H}$, $P_{\tilde{J}}^{⊥}=I_{K}-P_{\tilde{J}}$, $P_{\tilde{J}}=\tilde{J}{\left({\tilde{J}}^{H}\tilde{J}\right)}^{-1}{\tilde{J}}^{H}$, det(·) denotes the determinant of a square matrix.

### 3.2. Rao Test

To give the Rao test, we first need the concept of Fisher information matrix (FIM), defined as [23]

(5) $$I\left(\Theta \right)=E\left[\frac{\partial \ln f(X;\Theta )}{\partial \Theta^{*}}\frac{\partial \ln f(X;\Theta )}{\partial \Theta^{T}}\right],$$

where Θ stands for the unknown parameter set, E(·), ∂(·), ${\left(\cdot \right)}^{*}$, and ${\left(\cdot \right)}^{T}$ denote statistical expectation, partial derivative, conjugate, and transpose, respectively. The unknown parameter set is usually partitioned as $\Theta ={\left[\Theta_{r}^{T},\Theta_{s}^{T}\right]}^{T}$, where $\Theta_{r}$ and $\Theta_{s}$ are the related parameters and the nuisance parameters, respectively. Then, the FIM is partitioned as

(6) $$I\left(\Theta \right)=\left[\begin{array}{cc} I_{r,r}\left(\Theta \right) & I_{r,s}\left(\Theta \right)\\ I_{s,r}\left(\Theta \right) & I_{s,s}\left(\Theta \right) \end{array}\right].$$

The Rao test for complex-valued parameters is [23]

(7) $$t_{\mathrm{Rao}}={\left.\frac{\partial \ln f_{1}\left(X;\Theta \right)}{\partial \Theta_{r}}\right|}_{\Theta ={\hat{\Theta }}_{0}}^{T}{\left[I^{-1}\left({\hat{\Theta }}_{0}\right)\right]}_{r,r}{\left.\frac{\partial \ln f_{1}\left(X;\Theta \right)}{\partial \Theta_{r}^{*}}\right|}_{\Theta ={\hat{\Theta }}_{0}},$$

where $\Theta_{r}={\mathrm{vec}}^{T}\left(B\right)$, $\Theta_{s}=\left[\Theta_{s_{1}}^{T},\Theta_{s_{2}}^{T}\right]={\left[{\mathrm{vec}}^{T}\left(D\right),{\mathrm{vec}}^{T}\left(R\right)\right]}^{T}$, the notation vec(·) denotes the vectorization, ${\hat{\Theta }}_{0}$ is the maximum likelihood estimate (MLE) of Θ under hypothesis $H_{0}$, and

(8) $${\left[I^{-1}\left(\Theta \right)\right]}_{r,r}={\left[I_{r,r}\left(\Theta \right)-I_{r,s}\left(\Theta \right)\;I_{s,s}^{-1}\left(\Theta \right)\;I_{s,r}\left(\Theta \right)\right]}^{-1}.$$

In the following we successively derive the sub-matrices for the FIMs in (8). The joint probability density function (PDF) of X and $X_{M}$ under hypothesis $H_{1}$ is

(9) $$f_{1}\left(X,X_{M}\right)=\frac{\exp\left[-\mathrm{tr}\left(R^{-1}S\right)-\mathrm{tr}(R^{-1}X_{1}X_{1}^{H})\right]}{\pi^{N(K+M)}\det{\left(R\right)}^{K+M}},$$

where $X_{1}=X-AB-JD$. Taking the partial derivative of the logarithm of (9) with respect to vec(B) and $\mathrm{vec}(B^{*})$, we have

(10) $$\frac{\partial \;\ln f_{1}\left(X,X_{M}\right)}{\partial \;\mathrm{vec}(B)}=\mathrm{vec}\left(A^{T}R^{-T}X_{1}^{*}\right)$$

(11) $$\frac{\partial \;\ln f_{1}\left(X,X_{M}\right)}{\partial \;\mathrm{vec}(B^{*})}=\mathrm{vec}\left(A^{H}R^{-1}X_{1}\right).$$

Substituting (10) and (11) into (5), we have

(12) $$\begin{array}{cc} I_{r,r}\left(\Theta \right) & =E\left\{\mathrm{vec}\left(A^{H}R^{-1}X_{1}\right){\left[\mathrm{vec}\left(A^{T}R^{-T}X_{1}^{*}\right)\right]}^{T}\right\}\\ \; & =E\left\{\mathrm{vec}\left(A^{H}R^{-1}X_{1}\right){\left[I_{K}⊗\left(A^{T}R^{-T}\right)\mathrm{vec}\left(X_{1}^{*}\right)\right]}^{T}\right\}\\ \; & =\left(I_{K}⊗A^{H}R^{-1}\right)E\left[\mathrm{vec}\left(X_{1}\right)\mathrm{vec}{}^{H}\left(X_{1}\right)\right]\;\left(I_{K}⊗R^{-1}A\right)\\ \; & =I_{K}⊗\left(A^{H}R^{-1}A\right), \end{array}$$

where in the second equality we have used $\mathrm{vec}\left(F_{1}F_{2}F_{3}\right)=\left(F_{3}^{T}⊗F_{1}\right)\mathrm{vec}\left(F_{2}\right)$ for comparable matrices $F_{1}$, $F_{2}$, and $F_{3}$.

Taking the partial derivative of the logarithm of (9) with respect to vec(D) and $\mathrm{vec}(D^{*})$, we have the following two equalities

(13) $$\frac{\partial \;\ln f_{1}\left(X,X_{M}\right)}{\partial \;\mathrm{vec}(D)}=\mathrm{vec}\left(J^{T}R^{-T}X_{1}^{*}\right)$$

(14) $$\frac{\partial \;\ln f_{1}\left(X,X_{M}\right)}{\partial \;\mathrm{vec}(D^{*})}=\mathrm{vec}\left(J^{H}R^{-1}X_{1}\right).$$

Using (11) and (13), we have

(15) $$\begin{array}{cc} I_{r,s_{1}}\left(\Theta \right) & =\left(I_{K}⊗A^{H}R^{-1}\right)E\left[\mathrm{vec}\left(X_{1}\right)\mathrm{vec}{}^{H}\left(X_{1}\right)\right]\;\left(I_{K}⊗R^{-1}J\right)\\ \; & =\left(I⊗A^{H}R^{-1}\right)\left(I_{K}⊗R\right)\left(I_{K}⊗R^{-1}A\right)\\ \; & =I_{K}⊗\left(A^{H}R^{-1}J\right). \end{array}$$

We can similarly obtain $I_{r_{1},s_{2}}\left(\Theta \right)=0$ and $I_{s_{1},s_{1}}\left(\Theta \right)=I_{K}⊗\left(J^{H}R^{-1}J\right)$. According to [16], we have

(16) $$\begin{array}{c} I_{s_{2},s_{2}}\left(\Theta \right)=\left(M+K\right)R^{-T}⊗R^{-1}. \end{array}$$

It follows

(17) $$\begin{array}{c} I_{r,s}\left(\Theta \right)=\left[I_{K}⊗\left(A^{H}R^{-1}J\right)\;\;\;0\right] \end{array}$$

(18) $$\begin{array}{c} I_{s,s}\left(\Theta \right)=\left[\begin{array}{cc} I_{K}⊗\left(J^{H}R^{-1}J\right) & 0\\ 0 & \left(K+M\right)R^{-T}⊗R^{-1} \end{array}\right]. \end{array}$$

Substituting (12), (17), and (18) into (8) results in

(19) $${\left[I^{-1}\left(\Theta \right)\right]}_{r,r}=I_{K}⊗{\left({\bar{A}}^{H}P_{\bar{J}}^{⊥}\bar{A}\right)}^{-1},$$

where $\bar{A}=R^{-\frac{1}{2}}A$, $\bar{J}=R^{-\frac{1}{2}}J$, $P_{\bar{J}}^{⊥}=I_{N}-P_{\bar{J}}$, and $P_{\bar{J}}=\bar{J}{\left({\bar{J}}^{H}\bar{J}\right)}^{-1}{\bar{J}}^{H}$.

Substituting (10) and (19) into (7) results in the Rao test for given R and D

(20) $$\begin{array}{cc} t_{\mathrm{Rao}} & ={\mathrm{vec}}^{T}\left(A^{T}R^{-T}X_{0}^{*}\right)\left[I_{K}⊗{\left({\bar{A}}^{H}P_{\bar{J}}^{⊥}\bar{A}\right)}^{-1}\right]\mathrm{vec}\left(A^{H}R^{-1}X_{0}\right)\\ \; & ={\mathrm{vec}}^{T}\left(A^{T}R^{-T}X_{0}^{*}\right)\mathrm{vec}\left[{\left({\bar{A}}^{H}P_{\bar{J}}^{⊥}\bar{A}\right)}^{-1}{\bar{A}}^{H}{\bar{X}}_{0}\right]\\ \; & =\mathrm{tr}\left[{\left(\bar{X}-\bar{J}D\right)}^{H}\bar{A}{\left({\bar{A}}^{H}P_{\bar{J}}^{⊥}\bar{A}\right)}^{-1}{\bar{A}}^{H}\left(\bar{X}-\bar{J}D\right)\right], \end{array}$$

where tr(·) denotes the trace of a square matrix. Setting (13) to be zero result the MLE of D under hypothesis $H_{0}$

(21) $$\hat{D}={\left(J^{T}R^{-T}J\right)}^{-1}J^{T}R^{-T}X.$$

Substituting (21) into (20) results in the Rao test for given R

(22) $$\begin{array}{c} t_{\mathrm{Rao}}=\mathrm{tr}\left[{\bar{X}}^{H}P_{\bar{J}}^{⊥}\bar{A}{\left({\bar{A}}^{H}P_{\bar{J}}^{⊥}\bar{A}\right)}^{-1}{\bar{A}}^{H}P_{\bar{J}}^{⊥}\bar{X}\right]=\mathrm{tr}\left({\bar{X}}^{H}P_{P_{\bar{J}}^{⊥}\bar{A}}\bar{X}\right), \end{array}$$

According to (1), we have $P_{P_{\bar{J}}^{⊥}\bar{A}}=P_{\bar{J}}^{⊥}$. Hence, we can re-express (22) as

(23) $$t_{\mathrm{Rao}}=\mathrm{tr}\left({\bar{X}}^{H}P_{\bar{J}}^{⊥}\bar{X}\right),$$

which can be extended as

(24) $$t_{\mathrm{Rao}}=\mathrm{tr}\left[X^{H}R^{-1}X-X^{H}R^{-1}J{\left(J^{H}R^{-1}J\right)}^{-1}J^{H}R^{-1}X\right].$$

It is straightforward to show that

(25) $${\hat{R}}_{0}=\frac{1}{M+K}S^{\frac{1}{2}}\left(I_{N}+P_{\tilde{J}}^{⊥}\tilde{X}{\tilde{X}}^{H}P_{\tilde{J}}^{⊥}\right)S^{\frac{1}{2}}.$$

Performing matrix inversion to (25) and dropping the constant results in

(26) $${\hat{R}}_{0}^{-1}=S^{-\frac{1}{2}}\left[I_{N}-P_{\tilde{J}}^{⊥}\tilde{X}{\left(I_{K}+{\tilde{X}}^{H}P_{\tilde{J}}^{⊥}\tilde{X}\right)}^{-1}{\tilde{X}}^{H}P_{\tilde{J}}^{⊥}\right]S^{-\frac{1}{2}}.$$

Substituting (26) into (24), we have the final Rao test

(27) $$t_{\mathrm{Rao}}=\mathrm{tr}\left[{\tilde{X}}^{H}\tilde{X}-{\tilde{X}}^{H}P_{\tilde{J}}^{⊥}\tilde{X}{\left(I_{K}+{\tilde{X}}^{H}P_{\tilde{J}}^{⊥}{\tilde{X}}^{H}\right)}^{-1}{\tilde{X}}^{H}P_{\tilde{J}}^{⊥}\tilde{X}\right].$$

### 3.3. Wald Test

The Wald test for complex-valued parameters is [23]

(28) $$t_{\mathrm{Wald}}={\left({\hat{\Theta }}_{r_{1}}-\Theta_{r_{0}}\right)}^{H}{\left\{{\left[I^{-1}\left({\hat{\Theta }}_{1}\right)\right]}_{r,r}\right\}}^{-1}\left({\hat{\Theta }}_{r_{1}}-\Theta_{r_{0}}\right)$$

where ${\hat{\Theta }}_{r_{1}}$, $\Theta_{r_{0}}$, and ${\hat{\Theta }}_{1}$ are the MLE of $\Theta_{r}$ under hypothesis $H_{1}$, the value of ${\hat{\Theta }}_{r}$ under hypothesis $H_{0}$, and the MLE of Θ under hypothesis $H_{1}$, respectively. In a manner similar to (8), we have

(29) $${\left\{{\left[I^{-1}\left(\Theta \right)\right]}_{r,r}\right\}}^{-1}=I_{K}⊗{\bar{A}}^{H}P_{\bar{J}}^{⊥}\bar{A}.$$

The quantity $X_{1}$ in (9) can be written as $X_{1}=X-CF$, where C=[A,J] and F=[B;D]. Then nulling the derivative of (9) with respect to F results in the MLE of F as

(30) $$\hat{F}={\left(F^{T}R^{-T}F\right)}^{-1}F^{T}R^{-T}X.$$

Hence, the MLE of B is the first *p* column of (30). In a manner similar to [15], we can derive the MLE of B as

(31) $$\hat{B}={\left({\bar{A}}^{H}P_{\bar{J}}^{⊥}\bar{A}\right)}^{-1}{\bar{A}}^{H}P_{\bar{J}}^{⊥}\bar{X}.$$

Substituting (29) and (31) into (28) results in the Wald test for given R

(32) $$\begin{array}{cc} t_{\mathrm{Wald}}= & {\mathrm{vec}}^{H}\left[{\left({\bar{A}}^{H}P_{\bar{J}}^{⊥}\bar{A}\right)}^{-1}{\bar{A}}^{H}P_{\bar{J}}^{⊥}\bar{X}\right]\left[I_{K}⊗{\bar{A}}^{H}P_{\bar{J}}^{⊥}\bar{A}\right]\\ \; & \cdot \mathrm{vec}[{\left({\bar{A}}^{H}P_{\bar{J}}^{⊥}\bar{A}\right)}^{-1}{\bar{A}}^{H}P_{\bar{J}}^{⊥}\bar{X}]\\ = & {\mathrm{vec}}^{H}\left[{\left({\bar{A}}^{H}P_{\bar{J}}^{⊥}\bar{A}\right)}^{-1}{\bar{A}}^{H}P_{\bar{J}}^{⊥}\bar{X}\right]\mathrm{vec}\left[{\bar{A}}^{H}P_{\bar{J}}^{⊥}\bar{X}\right]\\ = & \mathrm{tr}\left[{\bar{X}}^{H}P_{\bar{J}}^{⊥}\bar{A}{\left({\bar{A}}^{H}P_{\bar{J}}^{⊥}\bar{A}\right)}^{-1}{\bar{A}}^{H}P_{\bar{J}}^{⊥}\bar{X}\right], \end{array}$$

which can be extended as

(33) $$\begin{array}{cc} t_{\mathrm{Wald}}= & \mathrm{tr}\left\{X^{H}\left[R^{-1}-R^{-1}J{\left(J^{H}R^{-1}J\right)}^{-1}J^{H}R^{-1}\right]\right.\\ \; & \cdot A{\left[A^{H}R^{-1}A-A^{H}R^{-1}J{\left(J^{H}R^{-1}J\right)}^{-1}J^{H}R^{-1}A\right]}^{-1}\\ \; & \left.\cdot A^{H}\left[R^{-1}-R^{-1}J{\left(J^{H}R^{-1}J\right)}^{-1}J^{H}R^{-1}\right]X\right\}. \end{array}$$

To obtain the final Wald test, we need the MLE of R under hypothesis $H_{1}$. Similar to (25), we have

(34) $${\hat{R}}_{1}=\frac{1}{M+K}S^{\frac{1}{2}}\left(I_{N}+P_{\tilde{C}}^{⊥}\tilde{X}{\tilde{X}}^{H}P_{\tilde{C}}^{⊥}\right)S^{\frac{1}{2}}.$$

Performing matrix inversion to (34) and dropping the constant results in

(35) $${\hat{R}}_{1}^{-1}=S^{-\frac{1}{2}}\left[I_{N}-P_{\tilde{C}}^{⊥}\tilde{X}{\left(I_{K}+{\tilde{X}}^{H}P_{\tilde{C}}^{⊥}\tilde{X}\right)}^{-1}{\tilde{X}}^{H}P_{\tilde{C}}^{⊥}\right]S^{-\frac{1}{2}}.$$

Post-multiplying (35) by A and J results in

(36) $${\hat{R}}_{1}^{-1}A=S^{-1}A$$

and

(37) $${\hat{R}}_{1}^{-1}J=S^{-1}J,$$

respectively. Substituting these two equalities into (33) results in

(38) $$\begin{array}{c} t_{\mathrm{Wald}}=\mathrm{tr}\left[{\tilde{X}}^{H}P_{\tilde{J}}^{⊥}\tilde{A}{\left({\tilde{A}}^{H}P_{\tilde{J}}^{⊥}\tilde{A}\right)}^{-1}{\tilde{A}}^{H}P_{\tilde{J}}^{⊥}\tilde{X}\right]=\mathrm{tr}\left({\tilde{X}}^{H}P_{P_{\tilde{J}}^{⊥}\tilde{A}}\tilde{X}\right). \end{array}$$

It can be shown that $P_{P_{\tilde{J}}^{⊥}\tilde{A}}=P_{\tilde{J}}^{⊥}$. Hence, we can rewrite (38) as

(39) $$\begin{array}{c} t_{\mathrm{Wald}}=\mathrm{tr}\left({\tilde{X}}^{H}P_{\tilde{J}}^{⊥}\tilde{X}\right). \end{array}$$

## 4. Performance Comparison

In this section, we investigate the detection performance of the proposed detectors with the existing ones. We compare the detection performance of the proposed detectors with the GLRT (We choose the GLRT0 for comparison, because the GLRT is the most common criterion for detector design.) for the detection problem in (2) when the interference is not taken into consideration, which is given by [24,25]

(40) $$t_{\mathrm{GLRT}0}=\frac{\det(I_{K}+{\tilde{X}}^{H}\tilde{X})}{\det(I_{K}+{\tilde{X}}^{H}P_{\tilde{A}}^{⊥}\tilde{X})}.$$

For convenience, we denote the detector in (40) as GLRT0.

**Figure 1:**
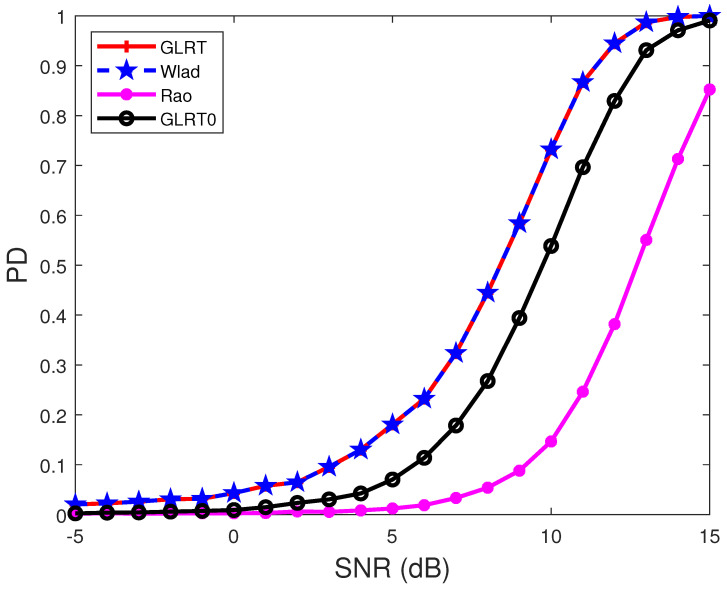
PDs of the detectors under different SNRs. $R\left(i_{1},i_{2}\right)=0.95^{|i_{1}-i_{2}|}$, N=8, p=1, M=2N, $\theta_{t}=0.2$, $\theta_{1}=0.25$, $\theta_{2}=0.4$, and INR = 5 dB.

**Figure 2:**
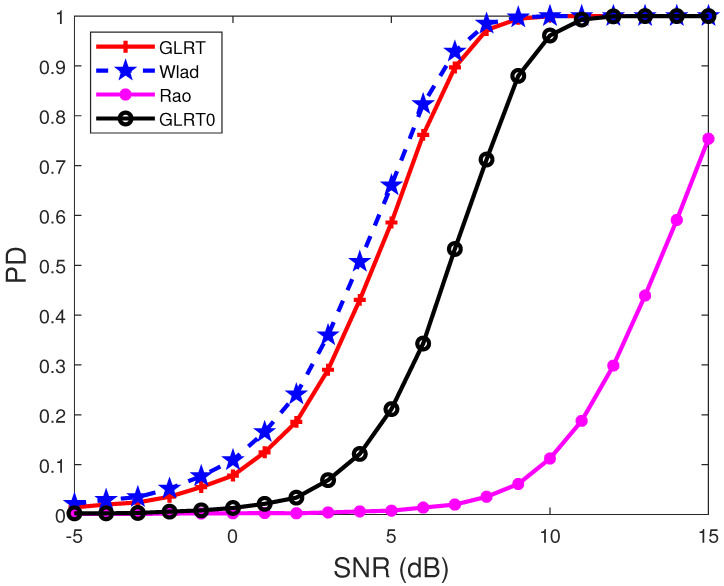
PDs of the detectors under different SNRs. $R\left(i_{1},i_{2}\right)=0.95^{|i_{1}-i_{2}|}$, N=8, p=2, M=2N, $\theta_{t_{1}}=-0.2$, $\theta_{t_{2}}=0.3$, $\theta_{1}=0.25$, $\theta_{2}=0.4$, and INR = 5 dB.

In Figure 1 and Figure 2, the noise covariance matrix is assumed to have the form $R\left(i_{1},i_{2}\right)=\sigma^{2}\rho^{|i_{1}-i_{2}|}$, $i_{1},i_{2}=1,2,\ldots ,N$, and we choose $\sigma^{2}=1$ and ρ=0.95. The signal-to-noise ratio (SNR) is defined as

(41) $$\mathrm{SNR}=\frac{1}{\sigma^{2}}\mathrm{tr}\left(B^{H}A^{H}AB\right).$$

Similarly, the interference-to-noise ratio (INR), when assuming the actual unknown interference, has the form $Q_{r}=J_{r}D_{r}$, which is defined as

(42) $$\mathrm{INR}=\frac{1}{\sigma^{2}}\mathrm{tr}(D_{r}^{H}J_{r}^{H}J_{r}D_{r}).$$

Figure 1 displays the PDs of the detectors under different SNRs when the target signal has the form $H=ab^{H}$,

(43) $$a={\left[1,e^{-j2\pi \theta_{t}},\ldots ,e^{-j2\pi \left(N-1\right)\theta_{t}}\right]}^{T},$$

$\theta_{t}$ is the normalized spatial frequency or normalized Doppler frequency, set to be $\theta_{t}=0.2$, and b is chosen to satisfy a certain SNR. Moreover, the unknown interference has the form $Q_{r}=J_{r}D_{r}$, where $J_{r}=\left[j_{1},j_{2}\right]$,

(44) $$j_{i}={\left[1,e^{-j2\pi \theta_{i}},\ldots ,e^{-j2\pi \left(N-1\right)\theta_{i}}\right]}^{T},$$

$\theta_{1}=0.25$, $\theta_{2}=0.4$, and $D_{r}$ is chosen to satisfy a given INR. The results show that the proposed GLRT and Wald test provide higher PDs than the GLRT0, which in turn has the higher PD than the Rao test. Moreover, the proposed GLRT has the same PD as the proposed Wald test for the chosen parameters. In fact, the GLRT is statistically equivalent to the Wald test when p=1. This is shown in Appendix A.

Figure 2 shows the PDs of the detectors under different SNRs when the signal has the form H=AB, $A=[a_{1},a_{2}]$,

(45) $$a_{i}={\left[1,e^{-j2\pi \theta_{t_{i}}},\ldots ,e^{-j2\pi \left(N-1\right)\theta_{t_{i}}}\right]}^{T},$$

$\theta_{t_{1}}=-0.2$, $\theta_{t_{2}}=0.3$, and the normalized interference angles are the same as those in Figure 1. The results indicate that the proposed Wald test has the highest PD, and the performance improvement in terms of SNR when PD = 0.8 is more than 2.5 dB, compared with the GLRT0. Moreover, comparing the results in Figure 1 and Figure 2 highlights that the PDs of the detectors in Figure 1 are lower than those in Figure 2. This is because the angles of the target and interference are closer in Figure 1 than in Figure 2.

**Figure 3:**
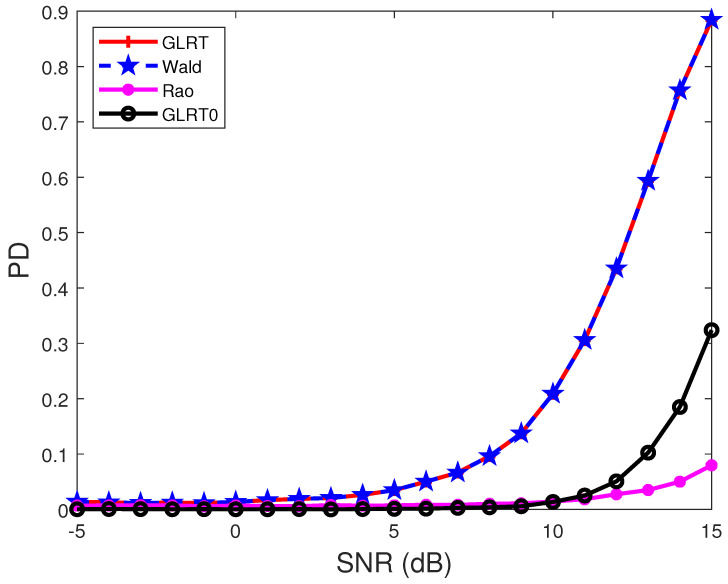
PDs of the detectors under different SNRs. $R\left(i_{1},i_{2}\right)=0.95^{|i_{1}-i_{2}|}e^{(i_{1}-i_{2})0.05}$, $\theta_{t}=0.2$, $\theta_{1}=0.25$, $\theta_{2}=0.3$, $\theta_{2}=0.4$, N=8, p=1, M=2N, and INR = 5 dB.

**Figure 4:**
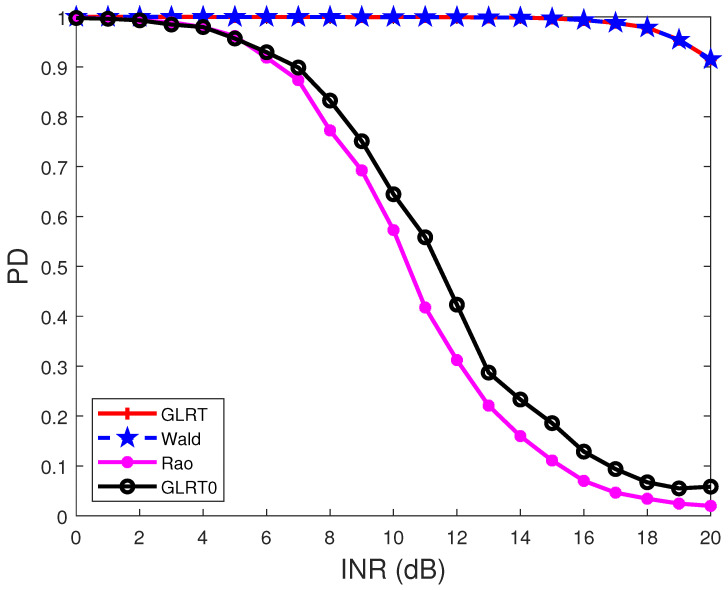
PDs of the detectors under different INRs. $R\left(i_{1},i_{2}\right)=0.95^{|i_{1}-i_{2}|}$, N=8, p=1, M=2N, $\theta_{t}=0.2$, $\theta_{1}=0.25$, $\theta_{2}=-0.2$, $\theta_{3}=0.4$, and SNR = 15 dB.

In Figure 3, the covariance matrix has the form $R\left(i_{1},i_{2}\right)=0.95^{|i_{1}-i_{2}|}e^{-\left(i_{1}-i_{2}\right)f_{d_{c}}}$, with $\sigma^{2}=0.95$ and $f_{d_{c}}=0.05$. This model of the covariance matrix can be taken as a generalization of that in Figure 1 and Figure 2, since the case of $f_{d_{c}}=0$ degenerates into the one adopted in Figure 1 and Figure 2. The results show that the proposed GLRT and the Wald test have much higher PDs than the GLRT0. Moreover, all the detectors suffer from performance loss for the chosen parameter setting, compared with the results in Figure 1 and Figure 2. Gathering the results in Figure 1, Figure 2, Figure 3 and Figure 4 indicates that the construction of the covariance matrix can affect the detection performance.

**Figure 5:**
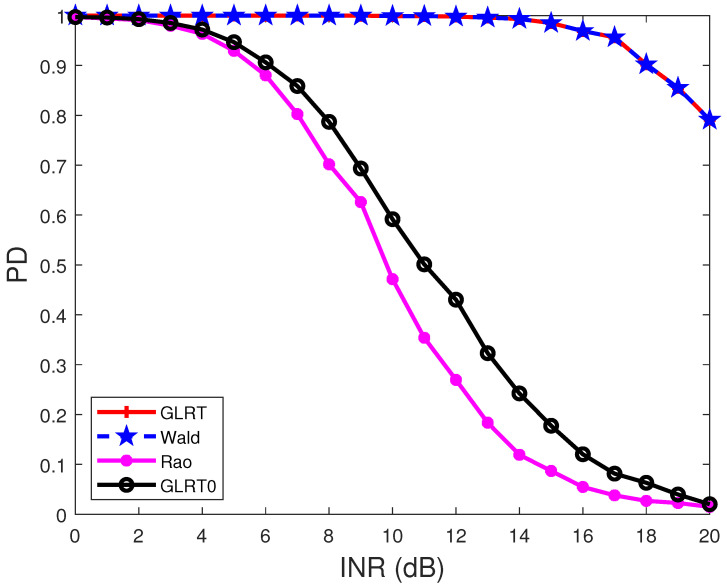
PDs of the detectors under different INRs. $R\left(i_{1},i_{2}\right)=0.95^{|i_{1}-i_{2}|}$, N=8, p=1, M=2N, $\theta_{t}=0.2$, $\theta_{1}=0.25$, $\theta_{2}=0.3$, $\theta_{3}=0.4$, and SNR = 15 dB.

Figure 4 and Figure 5 display the PDs of the detectors under different INRs. In Figure 4, the target normalized angle is $\theta_{t}=0.2$, and the interference normalized angles are $\theta_{1}=0.25$, $\theta_{2}=-0.2$, and $\theta_{3}=0.4$. In Figure 5, the target normalized angles are the same as those in Figure 4, while the interference normalized angles are $\theta_{1}=0.25$, $\theta_{2}=0.3$, and $\theta_{3}=0.4$. The results show that the PDs of the detectors decrease with the increase of the INR. Comparing the results in Figure 4 and Figure 5 indicates that when the interference is close to the target, the PD of a detector will decrease.

## 5. Conclusions

In this paper, we considered the problem of detecting a distributed target in unknown interference. To devise effective detectors, we temporally assumed that the interference is orthogonal to the signal subspace. Then, we proposed three detectors according to the GLRT, the Rao test, and the Wald test. An interesting finding is that the detection statistics of the three proposed detectors do not directly depend on the signal matrix. Instead, they depend on the matrix which is orthogonal to the signal matrix. Moreover, it is found that the GLRT coincides with the Wald test when the dimension of the signal subspace is equal to unity. Numerical examples show that all the three proposed detectors can effectively detect the target in the presence of unknown interference. The GLRT and the Wald test can achieve better detection performance than the existing detectors. 

## Data Availability

Not applicable.

## Appendix A. The Proof of the Equivalence between the GLRT and the Wald Test When *p* = 1

When p=1, the rank of the matrix $\tilde{J}$ is N−1, and hence, the rank of $P_{\tilde{J}}^{⊥}$ is one. Hence, we can express $P_{\tilde{J}}^{⊥}$ as $P_{\tilde{J}}^{⊥}=j_{v}j_{v}^{H}$, where $j_{v}$ is a proper N×1 vector. It follows that we can rewrite the GLRT in (4) as

(A1) $$t_{\mathrm{GLRT}}=\det\left(I_{K}+{\tilde{X}}^{H}j_{v}j_{v}^{H}\tilde{X}\right),$$

which can be recast as

(A2) $$t_{\mathrm{GLRT}}=1+j_{v}^{H}\tilde{X}{\tilde{X}}^{H}j_{v}.$$

Similarly, the Wald test when p=1 in (39) can be recast as

(A3) $$\begin{array}{c} t_{\mathrm{Wald}}=\mathrm{tr}\left({\tilde{X}}^{H}j_{v}j_{v}^{H}\tilde{X}\right)=j_{v}^{H}\tilde{X}{\tilde{X}}^{H}j_{v}, \end{array}$$

which, obviously, is equivalent to the above GLRT.
